# Draft Genome Sequence of *Xenophilus* sp., a Novel Bacterium Isolated from the Skin of a Southern Leopard Frog (*Rana sphenocephala*) in Florida, USA

**DOI:** 10.1128/genomeA.01067-17

**Published:** 2017-09-28

**Authors:** Brittany M. Lebert, Samantha A. Sanford, Levi M. Reisinger, Anna M. Forsman, Anna E. Savage

**Affiliations:** Department of Biology, University of Central Florida, Orlando, Florida, USA

## Abstract

We report here the draft genome sequence of a novel *Xenophilus* species cultured from the skin of a southern leopard frog (*Rana sphenocephala*). Compared to previously sequenced bacterial genomes, our novel isolate showed the most significant homology with *Xenophilus azovorans*. The assembled genome is 3,978,285 bp, with 3,704 predicted genes and one predicted plasmid.

## GENOME ANNOUNCEMENT

Massively parallel sequencing technologies are enabling the classification and characterization of novel bacterial diversity at unprecedented rates, and yet the majority of bacterial species remain undescribed. In this study, we contribute novel information to the database of bacterial genomes by sequencing a previously uncharacterized species cultured from the skin of a southern leopard frog (*Rana sphenocephala*). In August 2016, an *R. sphenocephala* individual was captured by a nitrile-gloved hand in a cypress dome wetland in Orlando, FL, rinsed with water, and placed briefly on a tryptic soy agar plate. After culturing overnight, a pure colony was picked from the plate, and DNA was extracted using a PowerSoil DNA isolation kit. After Nextera-style shotgun library preparation, genomic DNA was sequenced using the Illumina NextSeq 500 platform. PRINSEQ version 0.20.4 (1) was used to filter and trim raw reads after sequencing. Cleaned reads were assembled using SPAdes 3.9.1 (2). A second assembly was then performed using the *de novo* assembly feature in Geneious 10.0.5 (3) to assemble SPAdes contigs that had a minimum overlap of 99%.

BLASTn results for the 16S region of our unknown genome placed it into the family *Neisseriaceae* and the genus *Chromobacterium*. However, progressiveMauve alignments (4) between the unknown bacterial genome and all sequenced *Chromobacterium* species available in GenBank found little similarity. Consequently, we performed progressiveMauve alignments with the genomes from all sequenced *Neisseriaceae* species to attempt to find a suitable reference genome. The closest match, having the fewest locally collinear blocks (LCBs) with the highest weight, was the species *Hylemonella gracilis*. However, molecular phylogenetic analyses have moved *Hylemonella gracilis* (formerly classified as *Aquaspirillum gracile*) from *Neisseriaceae* to the family *Comamonadaceae* (5). Thus, our search for a reference genome was expanded to all genera in the family *Comamonadaceae*. Alignments were performed with one species from each genus with at least one sequenced genome available in GenBank, totaling 29 genome comparisons. After running all alignments, our unknown bacterial species was most similar to *Xenophilus azovorans* strain DSM 13620 (RefSeq accession number NZ_JQKD00000000). The alignment between the two genomes consisted of 2 LCBs with a weight of 619,350. *Xenophilus azovorans* is an aerobic bacterial species that has previously demonstrated the ability to break down azo dyes, which are common industrial byproducts (6). Close homology between our genome and *X. azovorans* allows for the classification of this previously unknown species into the genus *Xenophilus*. Because there is no evidence suggesting that this particular species has previously been sequenced, we classify it as a *Xenophilus* sp. The whole genome consisted of 3,978,285 bp, with a G+C content of 64.7%. There are 3,704 genes and one unknown plasmid. The addition of this novel *Xenophilus* sp. into the database of sequenced bacterial genomes gives new insight into both the genus *Xenophilus* and worldwide bacterial species diversity as a whole.

### Accession number(s).

This whole-genome shotgun project for *Xenophilus* sp. strain AP218F has been deposited at DDBJ/ENA/GenBank under the accession number NJIE00000000. The version described in this paper is version NJIE01000000.
